# Epidemiology of Salmonid Rickettsial Septicemia (SRS) in Farmed Salmon: The Role of Sea Lice Infestations in Mortality Risk

**DOI:** 10.1111/jfd.70097

**Published:** 2025-12-09

**Authors:** Benjamín Diethelm‐Varela, Nicolhole Atero, Francisca Córdova‐Bührle, Enrico L. Rezende, Stefan Gelcich, Osvaldo Sandoval, Carlos Navarro, Fernando O. Mardones

**Affiliations:** ^1^ Departamento de Genética Molecular y Microbiología, Facultad de Ciencias Biológicas Pontificia Universidad Católica de Chile Santiago Chile; ^2^ Escuela de Salud Pública Pontificia Universidad Católica de Chile Santiago Chile; ^3^ Dirección de Extensión y Vinculación con el Medio, Facultad de Ciencias Veterinarias y Pecuarias (FAVET) Universidad de Chile Santiago Chile; ^4^ Center of Applied Ecology and Sustainability (CAPES), Facultad de Ciencias Biológicas Pontificia Universidad Catolica de Chile Santiago Chile; ^5^ Instituto Milenio en Socioecologia Costera, Facultad de Ciencias Biológicas Pontificia Universidad Católica de Chile Santiago Chile; ^6^ Servicio Nacional de Pesca y Acuicultura, Ministerio de Economía, Gobierno de Chile Valparaíso Chile; ^7^ Escuela de Medicina Veterinaria, Facultad de Agronomía e Ingeniería Forestal, Facultad de Ciencias Biológicas y Facultad de Medicina Pontificia Universidad Católica de Chile Santiago Chile; ^8^ Division of Global Agriculture and Food Systems, Royal (Dick) School of Veterinary Studies The University of Edinburgh UK

**Keywords:** aquaculture, *Caligus rogercresseyi*, epidemiology, infectious diseases, *Piscirickettsia salmonis*, salmon farming

## Abstract

*Piscirickettsia salmonis* , the causal agent of salmonid rickettsial septicemia (SRS), is the main pathogen affecting farmed salmonids in Chile. Outbreaks of SRS lead to substantial economic losses for producers. Many determinants related to SRS outcomes are still poorly understood. Here, we conducted a retrospective cohort study from 2014 to 2021 to investigate the epidemiology of SRS at the farm level in southern Chile, employing historical monitoring data. Using time series analysis of weekly SRS mortality risk and sea lice ( *Caligus rogercresseyi* ) infestation levels, we found that SRS mortality risk had a strong seasonal component, with mortalities being significantly higher in the warmer seasons. While *Caligus* infestation levels have increased significantly over the years, SRS mortality risk has remained constant. Using mixed effects regression models, we identified that a key predictor for both increased weekly SRS mortality risk and higher hazard of reporting the first SRS outbreak of a production cycle was the level of female egg‐laying sea lice. We hypothesise that the interaction between sea lice, *P. salmonis* and rising water temperatures may produce synergistic stress on salmon that accelerates disease progression and prompts overuse of antimicrobials. This calls for an urgent integrated pest management approach in aquaculture practice.

## 1 Introduction

Aquaculture is a rapidly expanding economic activity poised to play an increasingly important role in ensuring global food security (Costello et al. 2020; Golden et al. 2021; Naylor et al. 2021). Within this sector, salmon farming (salmoniculture) represents a major component, with Norway and Chile leading global production by biomass (Naylor et al. 2021). Despite its potential, the industry faces significant challenges—foremost among them are management of infectious diseases, which pose major threats by causing substantial production losses, economic damages, poor fish welfare and food waste (Naylor et al. 2021; Stentiford et al. 2012).

Among the pathogens affecting farmed salmonids, *Piscirickettsia salmonis* stands out as a particularly concerning agent. *P. salmonis* is a Gram‐negative, non‐motile, facultative intracellular bacterium that causes salmonid rickettsial septicemia (SRS), also known as piscirickettsiosis. SRS is a systemic disease with a wide array of signs, including neurological, hepatic and neurological manifestations (Rozas and Enriquez 2014). First described in 1981 as a disease affecting farmed coho salmon ( *Oncorhynchus kisutch* ) in Chile (Bravo and Campos 1989), the pathogen also demonstrated pathogenicity against Atlantic salmon ( *Salmo salar* ), rainbow trout ( *O. mykiss* ) and Chinook salmon ( *Oncorhynchus tshawytscha* ) (Fryer et al. 1992; Mauel et al. 2008; Mikalsen et al. 2008; Schäfer et al. 1990; Yanez et al. 2016). While present in all major salmon‐producing countries, *P. salmonis* has reached a hyperendemic status in Chile, with farm‐level prevalence nearing 80% and annual losses exceeding USD 700 million (Maisey et al. 2017; Servicio Nacional de Pesca y Acuicultura 2012a). The underlying reasons for this high endemicity remain uncertain. The Chilean salmon industry employs several vaccines for SRS prevention; however, these products are generally characterised as delaying rather than preventing outbreaks (Rozas and Enriquez 2014; Valenzuela‐Aviles et al. 2022). Consequently, active outbreaks are primarily controlled through antibiotic treatments, most notably florfenicol, raising concerns regarding the emergence and spread of antimicrobial resistance (Avendaño‐Herrera et al. 2023; Rozas and Enriquez 2014). SRS is therefore arguably one of the most critical threats to the long‐term sustainability of the Chilean salmon farming industry.

In Chile, due to the significant impact of SRS on the salmon industry (Servicio Nacional de Pesca y Acuicultura 2006, 2011), a specific official surveillance programme has been in place since 2012 (Servicio Nacional de Pesca y Acuicultura 2012a). Established by the National Fisheries and Aquaculture Service (Sernapesca), the programme—known as the Specific Sanitary Surveillance and Control Program for Piscirickettsiosis (PSEVC‐Piscirickettsiosis)—aims to reduce the impact of *P. salmonis* through early detection, regular and risk‐based surveillance and timely implementation of control measures. The programme classifies aquaculture sites into risk categories (e.g., under surveillance, alert or high dissemination) based on disease indicators, and mandates action plans that include increased mortality removal, treatment and harvest strategies. It also requires regular reporting, laboratory confirmation and region‐specific preventive measures (Servicio Nacional de Pesca y Acuicultura 2012a).

In addition, the General Sanitary Program for Mortality Management (PSGM), also established in 2013 by Sernapesca (Servicio Nacional de Pesca y Acuicultura 2012b), standardises procedures for the classification, handling and safe disposal of fish mortalities in salmon farms to prevent the spread of high‐risk diseases. The programme mandates daily removal of mortalities, detailed classification of cause of death (including infectious and environmental causes) and appropriate disposal via composting, ensiling or incineration. It further requires regular reporting to Sernapesca, proper staff training and strict biosecurity measures to ensure containment and traceability throughout the mortality management process. Together, these programmes support the Integrated Aquaculture Surveillance System (SIFA), a comprehensive database that collects information on salmonid mortalities from all causes, diagnostic results, antimicrobial usage, parasite infestations and antiparasitic treatments, as reported by salmon farming companies.

Leveraging this centralised source of information, several epidemiological studies have been published analysing the Chilean salmon farming system (Arriagada et al. 2019; Bravo et al. 2020; Happold, Meyer, et al. 2020; Happold, Sadler, et al. 2020; Hillman et al. 2020; Jakob et al. 2014; Meyer et al. 2019, 2021; Price et al. 2016, 2017, 2020; Rees et al. 2014). These investigations span a wide range of research questions, methodological approaches and data sources. Among them, six studies have specifically utilised data from the SIFA database to study SRS. Arriagada et al. (2019) used SIFA data to assess the association between *Caligus* infestations and SRS mortalities, finding that the mean abundance of adult sea lice was linked to cumulative SRS mortality (Arriagada et al. 2019); however, the time range used in this study was limited (2014–2016), and the model constructed by the authors controlled for relatively few additional variables. Bravo et al. (2020) analysed water‐related variables influencing SRS prevalence and transmission, identifying the number of diseased farms and upstream SRS outbreaks as the strongest predictors (Bravo et al. 2020). In the same year, Price et al. (2020) evaluated the sampling and surveillance strategies in place and concluded that they are effective in mitigating SRS outbreaks. Hillman et al. (2020) conducted a retrospective study covering 2011–2017 and found that the risk of SRS mortality was significantly associated with the salmonid species (Hillman et al. 2020). Meyer et al. (2019) investigated the effects of sea lice burden and delousing treatments, showing that both were significant risk factors for increased SRS mortality (Meyer et al. 2019). Later, in 2021, the same research team modelled the spatiotemporal autocorrelation of SRS mortality risk across the Chilean salmon farming system (Meyer et al. 2021). The primary methodological approaches employed in these studies include generalised linear models with mixed effects and survival analysis (Arriagada et al. 2019; Bravo et al. 2020; Hillman et al. 2020; Meyer et al. 2019, 2021; Price et al. 2020).

Despite the existence of the national SRS surveillance system and important research advances, as described above, significant gaps remain in the management of the disease, chief among them is the limited understanding of the factors that determine the severity of SRS outbreaks. In this context, systematising existing knowledge on SRS outbreaks in farmed salmon is essential. Estévez et al. (2019) published a study in which a panel of experts identified and ranked 17 potential predictors of SRS mortality, drawing from both industry and academic perspectives. This work provided a foundation for a more integrated, data‐driven approach to analysing the impact of these predictors (Estévez et al. 2019). Building on that contribution, the present study consists of a retrospective cohort analysis of SRS mortality in Chile, using mandatory reporting data archived in the SIFA database. Our objectives are to produce a comprehensive, historical analysis employing a unified methodological framework to improve the understanding of SRS epidemiology and to identify predictors of SRS risk that may serve as actionable tools for better disease management, with a special emphasis on *Caligus* infestations. To achieve these goals, we evaluate a set of potential risk factors from the salmon farming system and use generalised linear models and survival analysis to investigate two key epidemiological outcomes: the weekly risk of SRS mortality during a production cycle and the time to the first SRS outbreak within a cycle.

## 2 Materials and Methods

### 2.1 Data Source and Study Unit

Sernapesca compiles all salmonid mortalities in Chile on a cage‐by‐cage, per‐farm, per‐epidemiological week basis in its SIFA database. This dataset consists of mortalities from both infectious and non‐infectious causes. Each weekly observation is linked to a specific farm code and a farming area defined by Sernapesca (referred to as ‘neighbourhood’ or ‘ACS’ (Spanish acronym) in industry terminology). The Service also keeps, in different datasets, records of *Caligus* infestation reports. Since these are also aggregated on a per‐farm‐per‐week basis, we were able to merge the mortalities and *Caligus* reports datasets using the week and farm code information. Our merged dataset ranged from June 2014 to March 2021 and includes data from 652 salmon farms grouped across 59 farming neighbourhoods. In our merged dataset, each observation corresponds to an epidemiological week of a specific production cycle in a particular salmon farm.

### 2.2 Study Design and Data Management

We conducted a retrospective cohort study using the merged Sernapesca dataset, including all salmon farms in the Los Lagos and Aysén regions of Chile—areas where SRS has been reported (Servicio Nacional de Pesca y Acuicultura 2015, 2021). Farms in the Magallanes region were excluded, as the region is considered free of SRS (Servicio Nacional de Pesca y Acuicultura 2015, 2021). The salmon farm served as the epidemiological unit of analysis. Production cycles were annotated in the data using an R programming script (see Supporting Information S1); start and end dates of the cycles are discernible because of gaps of 2 weeks or longer in the data report for a specific farm. This approach to cycle annotation was based on previous research (Hillman et al. 2020).

SRS mortality risk for a given period was defined as the proportion of deaths attributed by on‐site veterinarians to SRS, divided by the median farm population over that period. We defined the start of an SRS outbreak as the first week of a 3‐week window in which the average SRS mortality risk equalled or exceeded 0.05%. Outbreaks were considered to end in the first week of a 3‐week period where the combined average mortality risk due to SRS and unknown causes dropped below 0.1%. These thresholds are based on previous studies (Arriagada et al. 2019; Happold, Meyer, et al. 2020; Happold, Sadler, et al. 2020; Jakob et al. 2014). For each outbreak, the post‐outbreak SRS mortality risk was calculated as the proportion of SRS‐attributed deaths over the median farm population between outbreak start and end.

### 2.3 Descriptive Epidemiology and Time Series Analyses

Descriptive analyses were conducted to characterise the epidemiology of SRS and other mortality causes in southern Chile during the study period (June 2014 to March 2021). Descriptive statistics, charts and maps were used to summarise key variables, including SRS mortality risk, mortality from other causes, *Caligus* infestation levels, seasonality and farm geographic distribution. To complement these analyses and explore temporal patterns, we conducted time series analyses aimed at evaluating long‐term trends and seasonal fluctuations in SRS mortality risk, as well as in *Caligus* infestations. Given the incomplete periodicity observed in the weekly data, we aggregated the data at the monthly level for time series evaluation. Trend and seasonality analyses were performed using classical time series statistical methods and were further supported by linear mixed‐effects models to account for hierarchical structure in the data.

### 2.4 Outcomes and Putative Predictors

This study focused on two primary outcome variables: the weekly SRS mortality risk per farm and the time until the first SRS outbreak occurs within a production cycle. A range of putative predictors were considered potential risks or protective factors for these outcomes. These included environmental and geographical variables, such as latitude, longitude and season, as well as salmonid species. Outbreaks of infectious diseases—including heart and skeletal muscle inflammation (HSMI), tenacibaculosis, amoebiasis and infectious pancreatic necrosis (IPN)—were also included as predictors. Additionally, we assessed the impact of high mortality events due to non‐infectious causes, including mechanical damage, predator attacks, poor adaptation following seawater transfer, deformities, environmental stress and the culling of unfit or diseased animals. Variables related to *Caligus* infestations were incorporated, including the rate of increase in infestation load over a 3‐week period, and the occurrence of high‐load events. To account for the hierarchical structure of the data, clustering variables were included as random effects in the models. These variables comprise identifiers which allow assigning each observation into a specific cluster of observations which, because of their belonging to a hierarchical structure, comprise pseudo‐replicates. Such clustering variables consist of the ACS, the unique farm identification code and the specific production cycle. A full list of all variables and their categories is presented in Table 1. Some mortality causes—such as mycosis and flavobacteriosis—were excluded from the analysis due to their relevance primarily in freshwater systems (Servicio Nacional de Pesca y Acuicultura 2023a). Similarly, trout idiopathic syndrome and icteric syndrome were excluded because of their species‐specific nature (Servicio Nacional de Pesca y Acuicultura 2023a). The selection of mortality‐related variables was based on epidemiological relevance as defined in Sernapesca reports (Servicio Nacional de Pesca y Acuicultura 2023b).

**TABLE 1:** Putative predictors for SRS mortality risk, grouped by categories, using retrospective data from 652 salmon farms in southern Chile from June 2014 to March 2021.

Category	Variables	Variable type
Geographical and environmental variables	Latitude (degrees)	Continuous
	Longitude (degrees)	Continuous
	Season	Nominal
Salmonid species	Salmonid species	Nominal
Mortalities due to infectious diseases	Tenacibaculosis outbreak^a^	Count
	Bacterial kidney disease (BKD) outbreak	Count
	Heart skeletal muscle inflammation (HSMI) outbreak	Count
	Amoebiasis outbreak	Count
	Infectious pancreatic necrosis (IPN) outbreak	Count
Mortalities due to other causes	Predator attack‐related high‐mortality events^b^	Count
	Environmental‐related high‐mortality events	Count
	Elimination‐related high‐mortality events	Count
	Mechanical damage‐related high‐mortality events	Count
	Lack of adaptation‐related high‐mortality events	Count
	Deformity‐related high‐mortality events	Count
	Maturity‐related high‐mortality events	Count
*Caligus* variables	Positive growth rate^c^ of the *Caligus* average infestation load^d^ over a 3‐week period	Dichotomous
	High load *Caligus* report^e^	Count
Clustering variables (used as random effects)	Salmoniculture ‘neighbourhood’ identification code	Nominal
	Farm identification code	Nominal
	Production cycle identification code	Nominal

^a^ Outbreaks of an infectious disease are defined as a period starting on the first week of a 3‐week period where the weekly mortality risk of the infectious disease exceeds 0.05% and ending on the first week of a 3‐week period where the mortality risk of the infectious disease plus unknown causes is less than 0.1%.

^b^ High‐mortality events of a non‐infectious cause are defined as a period starting in the first week of a 3‐week period where the weekly mortality risk from the non‐infectious cause exceeds 0.05% and ending in the first week of a 3‐week period where the mortality risk from the non‐infectious cause plus unknown causes is less than 0.1%.

^c^ A positive growth rate is defined as a situation where the linear regression slope of the infestation load values over a 3‐week period is greater than zero.

^d^ The average infestation load is defined as the mean count of female egg‐laying parasites across a sample of 10 salmon in a week. Egg‐laying female parasites are adult female parasites that carry egg sacs and are usually employed as the *Caligus* counting unit.

^e^ A high‐load *Caligus* report is defined as a weekly *Caligus* report where the average infestation load is greater than 1 female egg‐laying parasite per salmon. This criterion is used by Sernapesca to identify a ‘high‐dissemination farm’.

### 2.5 Statistical Modelling

Two main explanatory models were developed for this study. The first model evaluated weekly SRS mortality risk using a mixed‐effects negative binomial regression, implemented with the glmmTMB package in R. Time, defined as the number of weeks since the beginning of the production cycle, was included as a continuous fixed effect. Weekly SRS mortality counts were modelled as the dependent variable, with the logarithm of the average farm population included as an offset to model mortality risk appropriately. Predictor variables, as detailed in Table 1, were included in the model selection process. The second model assessed the time to the first SRS outbreak using a Cox proportional hazards regression with frailty terms to account for random effects, implemented via the coxme package. Like the first model, it was adjusted using selected predictors and clustering variables. To capture temporal dynamics, infectious and non‐infectious mortality causes were incorporated differently across models. In the negative binomial model, these were entered as dichotomous variables indicating whether the current week occurred before or after the onset of a corresponding outbreak or high‐mortality event. In the Cox model, they were entered as count variables, reflecting the number of such events observed prior to the first SRS outbreak (or prior to the end of the production cycle in non‐outbreak cases). All numerical predictors were standardised (mean = 0, standard deviation = 1) prior to analysis. Model construction involved fitting both null and maximal models, followed by a manual backward selection process in which the least significant variables were removed iteratively. Predictors were retained in the final model based on a significance threshold of *p* < 0.05. The Akaike Information Criterion (AIC) was used throughout to identify the model with the best fit. All data management, statistical analysis and visualisations were performed using the R programming language, employing the packages *glmmTMB, coxme, dplyr and janitor (Brooks et al. 2017; Firke 2016; R Core Team 2023; Therneau 2009; Wickham et al. 2014).

## 3 Results

### 3.1 Descriptive Epidemiology

The final dataset included 1546 production cycles from 652 salmon farms distributed across 59 ACS zones (Table 2). The median duration of production cycles across all species was 63 weeks, with notable interspecies differences. Atlantic salmon exhibited the longest production cycles with a median duration of 71 weeks, followed by rainbow trout at 45 weeks and coho salmon at 41 weeks. The overall median weekly SRS mortality risk across all cycles was 0.0022 deaths per 1000 fish, although this varied considerably by species. Atlantic salmon showed the highest median weekly SRS mortality risk at 0.015 deaths per 1000 fish. In contrast, both rainbow trout and coho salmon exhibited negligible risk, each with a median of zero deaths per 1000 fish.

**TABLE 2:** Descriptive epidemiology of the SIFA dataset of the Chilean aquaculture throughout the 2014–2021 period.

Variable	All species	Atlantic salmon	Rainbow trout	Coho salmon
Cycle duration (median (min‐max))	63 (27–94)	71 (28–94)	45 (29–68)	41 (27–71)
Weekly SRS mortality risk (deaths per 1000 animals) (median (min‐max))	0.0022 (0–130)	0.015 (0–110)	0 (0–130)	0 (0–67)
Summer weekly SRS mortality risk (deaths per 1000 animals) (median (min‐max))	0.022 (0–110)	0.052 (0–110)	0.0076 (0–56)	0 (0–14)
Fall weekly SRS mortality risk (deaths per 1000 animals) (median (min‐max))	0.0042 (0–130)	0.03 (0–85)	0.0014 (0–130)	0 (0–7.5)
Winter weekly SRS mortality risk (deaths per 1000 animals) (median (min‐max))	0 (0–83)	0.0017 (0–71)	0 (0–18)	0 (0–11)
Spring weekly SRS mortality risk (deaths per 1000 animals) (median (min‐max))	0 (0–71)	0.0061 (0–77)	0 (0–83)	0 (0–67)
Cycles where a high‐load *Caligus* report was experienced (count (%))	1546 (62)	955 (89)	179 (60)	412 (0)
Experienced at least one SRS outbreak (count (%))	1546 (60)	955 (77)	179 (69)	412 (17)
Number of SRS outbreaks during the cycle (median (min‐max))	1 (0–5)	1 (0–5)	1 (0–3)	0 (0–2)
SRS outbreak duration in weeks (mean (SD))	6.3 (5.2)	6 (4.8)	9.4 (6.8)	6.2 (5.4)
Number of tenacibaculosis outbreaks during the cycle (median (min‐max))	0 (0–4)	0 (0–4)	0 (0–2)	0 (0–3)
Tenacibaculosis outbreak duration in weeks (mean (SD))	6 (6.2)	6.2 (6.4)	5.5 (3.8)	4.5 (5)
Number of BKD outbreaks during the cycle (median (min‐max))	0 (0–3)	0 (0–2)	0 (0–0)	0 (0–3)
BKD outbreak duration in weeks (mean (SD))	5.8 (5.1)	4.8 (3)	0 (0)	7.1 (6.7)
Number of HSMI outbreaks during the cycle (median (min‐max))	0 (0–2)	0 (0–1)	0 (0–0)	0 (0–2)
HSMI outbreak duration in weeks (mean (SD))	6.2 (3.9)	6 (3.6)	0 (0)	6.6 (4.7)
Number of amoebiasis outbreaks during the cycle (median (min‐max))	0 (0–2)	0 (0–1)	0 (0–0)	0 (0–2)
Amoebiasis outbreak duration in weeks (mean (SD))	6.3 (5)	6.9 (5.4)	2.4 (0.89)	4.9 (2)
Number of IPN outbreaks during the cycle (median (min‐max))	0 (0–2)	0 (0–2)	0 (0–1)	0 (0–1)
IPN outbreak duration in weeks (mean (SD))	4.8 (2.6)	4.8 (2.6)	5.3 (1.2)	6 (0)
Number of predator attack‐related high mortality during the cycle (median (min‐max))	0 (0–6)	1 (0–6)	1 (0–5)	0 (0–4)
Predator attack high‐mortality event duration in weeks (mean (SD))	4.8 (4.6)	4.8 (4.5)	5.3 (5.5)	4.5 (3.7)
Number of environmental‐related high mortality during the cycle (median (min‐max))	0 (0–5)	1 (0–5)	0 (0–4)	0 (0–3)
Environmental high‐mortality event duration in weeks (mean (SD))	4.5 (3.2)	4.5 (3.2)	4.7 (3.8)	3.9 (2.4)
Number of elimination‐related high mortality during the cycle (median (min‐max))	0 (0–5)	0 (0–5)	1 (0–4)	0 (0–4)
Elimination high‐mortality event duration in weeks (mean (SD))	4.8 (3.5)	4.3 (2.7)	6.5 (5.4)	5.2 (6.5)
Number of mechanical damage‐related high mortality during the cycle (median (min‐max))	1 (0–9)	2 (0–9)	1 (0–5)	0 (0–6)
Mechanical damage high‐mortality event duration in weeks (mean (SD))	4.5 (4.5)	4.6 (4.6)	4 (4.3)	3.8 (3.6)
Number of lack of adaptation‐related high mortality during the cycle (median (min‐max))	1 (0–6)	1 (0–6)	1 (0–4)	1 (0–5)
Lack of adaptation high‐mortality event duration in weeks (mean (SD))	6.5 (6.9)	5.8 (6.4)	6.8 (6.7)	8 (7.8)
Number of deformity‐related high mortality during the cycle (median (min‐max))	0 (0–5)	0 (0–5)	0 (0–3)	0 (0–3)
Deformity‐related high‐mortality event duration in weeks (mean (SD))	4.6 (4.9)	3.3 (2.3)	4.5 (3.8)	6.2 (6.6)
Number of maturity‐related high mortality during the cycle (median (min‐max))	0 (0–4)	0 (0–4)	0 (0–2)	0 (0–2)
Maturity‐related high‐mortality‐event duration in weeks (mean (SD))	6.2 (5.3)	6.2 (5.4)	5.2 (4)	6.7 (5.7)
Latitude (degrees) (median (min‐max))	43 (41–46)	44 (41–46)	42 (41–46)	42 (41–46)
Longitude (degrees) (median (min‐max))	73 (72–74)	73 (72–74)	73 (72–74)	73 (72–74)

SRS mortality risk displayed strong seasonal variation. The highest risks were observed during the summer months, when the overall median weekly mortality risk reached 0.022 deaths per 1000 fish. This seasonal peak was predominantly driven by Atlantic salmon, which had a median summer risk of 0.052 deaths per 1000 fish. Rainbow trout showed a lower but measurable summer risk of 0.0076 deaths per 1000 fish, while coho salmon continued to show negligible risk. During fall, winter and spring, mortality risks declined sharply. In winter, median weekly SRS mortality risk was close to zero for all species, with only Atlantic salmon showing a measurable risk of 0.0017 deaths per 1000 fish. Across fall and spring, the median mortality risk remained approximately zero for all species.

High‐load *Caligus* reports were observed in 62% of all production cycles. When broken down by species, 89% of Atlantic salmon cycles reported high‐load *Caligus* events, compared to 60% of rainbow trout cycles and 0% of coho salmon cycles.

Of the 1546 production cycles analysed, 60% experienced at least one SRS outbreak. Among species, 77% of Atlantic salmon cycles reported at least one outbreak, followed by 69% of rainbow trout cycles and 17% of coho salmon cycles. The median number of SRS outbreaks per cycle was one, with Atlantic salmon and rainbow trout both showing a median of one outbreak per cycle, while coho salmon cycles had a median of zero.

Additional descriptive statistics related to mortality causes other than SRS, as well as geographic distribution (latitude and longitude), are provided in Table 2.

### 3.2 Time Series Analyses

**FIGURE 1:**
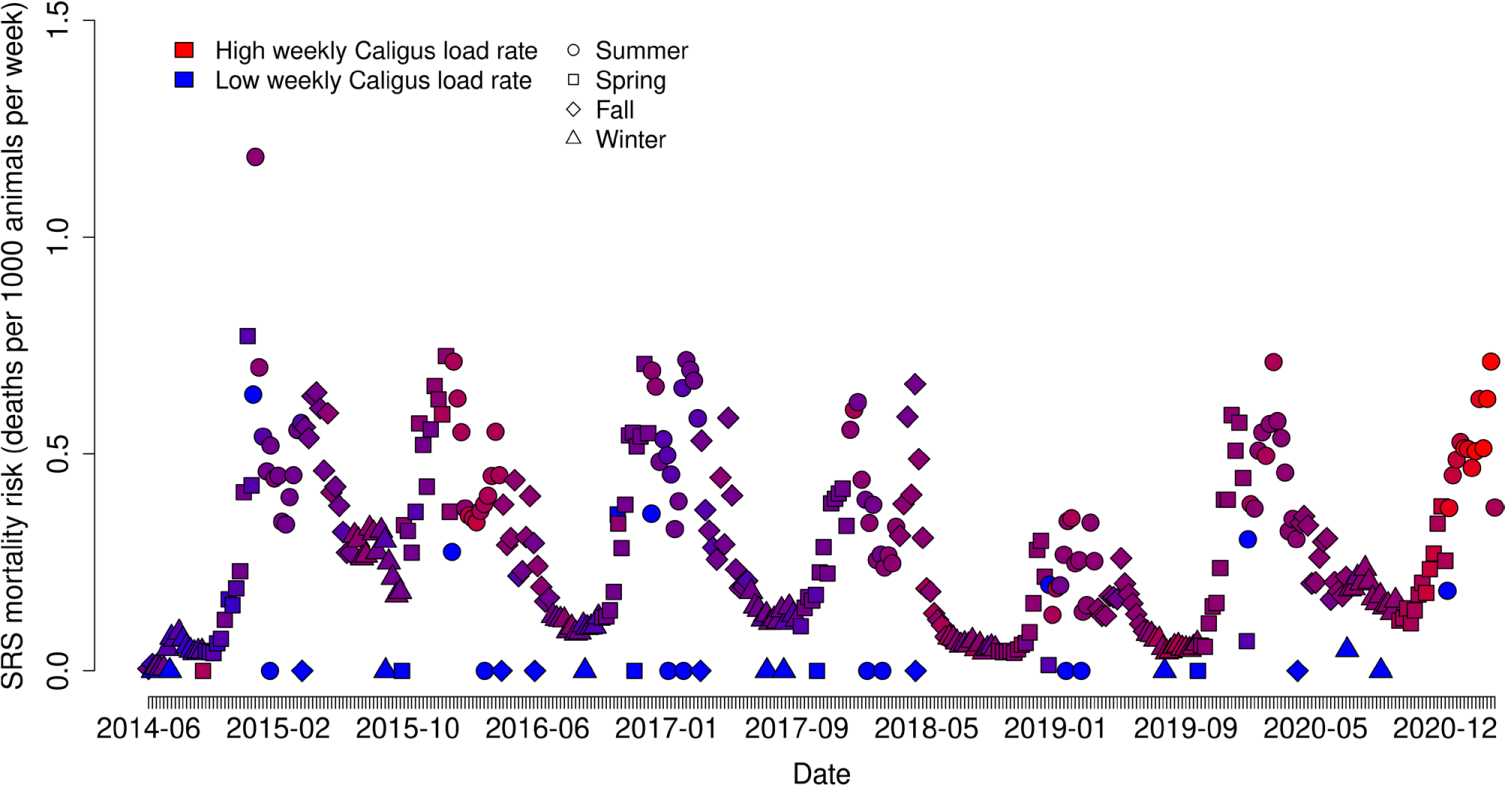
Time series of SRS mortality risk in the Chilean salmoniculture system in the Los Lagos and Aysén regions between the years 2014 and 2021. Each datapoint corresponds to an epidemiological week within a production cycle. Blue datapoints along the bottom of the y‐axis indicate epidemiological weeks where SRS mortality risk was at or near zero, and Caligus infestation loads were low.

The temporal dynamics of SRS mortality across the entire study period are presented in Figure 1. Time series of other mortality causes are provided in the Supporting Information S1 (Figure S1 for infectious diseases and Figure S2 for non‐infectious causes). To evaluate trends and seasonal patterns, we performed time series analyses of SRS mortality risk and *Caligus* infestation loads. These analyses were complemented by linear mixed‐effects models using the same variables to quantify seasonal effects and long‐term trends. The decomposition of the SRS mortality risk time series revealed a clear seasonal pattern, as supported by visual inspection (Figure S3) and confirmed by linear mixed‐effects modelling (Table S1). No significant long‐term trend was observed in SRS mortality risk across the study period, a conclusion supported by visual inspection (Figure S3) and by the results of the Augmented Dickey‐Fuller test (test statistic = −5.42, *p*‐value = 0.01), which rejected the null hypothesis of non‐stationarity, and by linear mixed‐effects regression (Table S2), indicating that the SRS mortality risk remained relatively stable over time.

In contrast, the time series decomposition of *Caligus* infestation loads revealed both seasonal variation and an increasing long‐term trend. Seasonal fluctuations were evident through visual inspection (Figure S4) and supported by mixed‐effects modelling (Table S3). The long‐term trend was found to be non‐stationary, as indicated by the Augmented Dickey‐Fuller test, which failed to reject the null hypothesis of non‐stationarity (test statistic = −1.28, *p*‐value = 0.87). This finding was further corroborated by linear mixed‐effects analysis (Table S4), suggesting a gradual increase in *Caligus* infestation loads over the course of the study period.

### 3.3 SRS Weekly Mortality Risk

**FIGURE 2:**
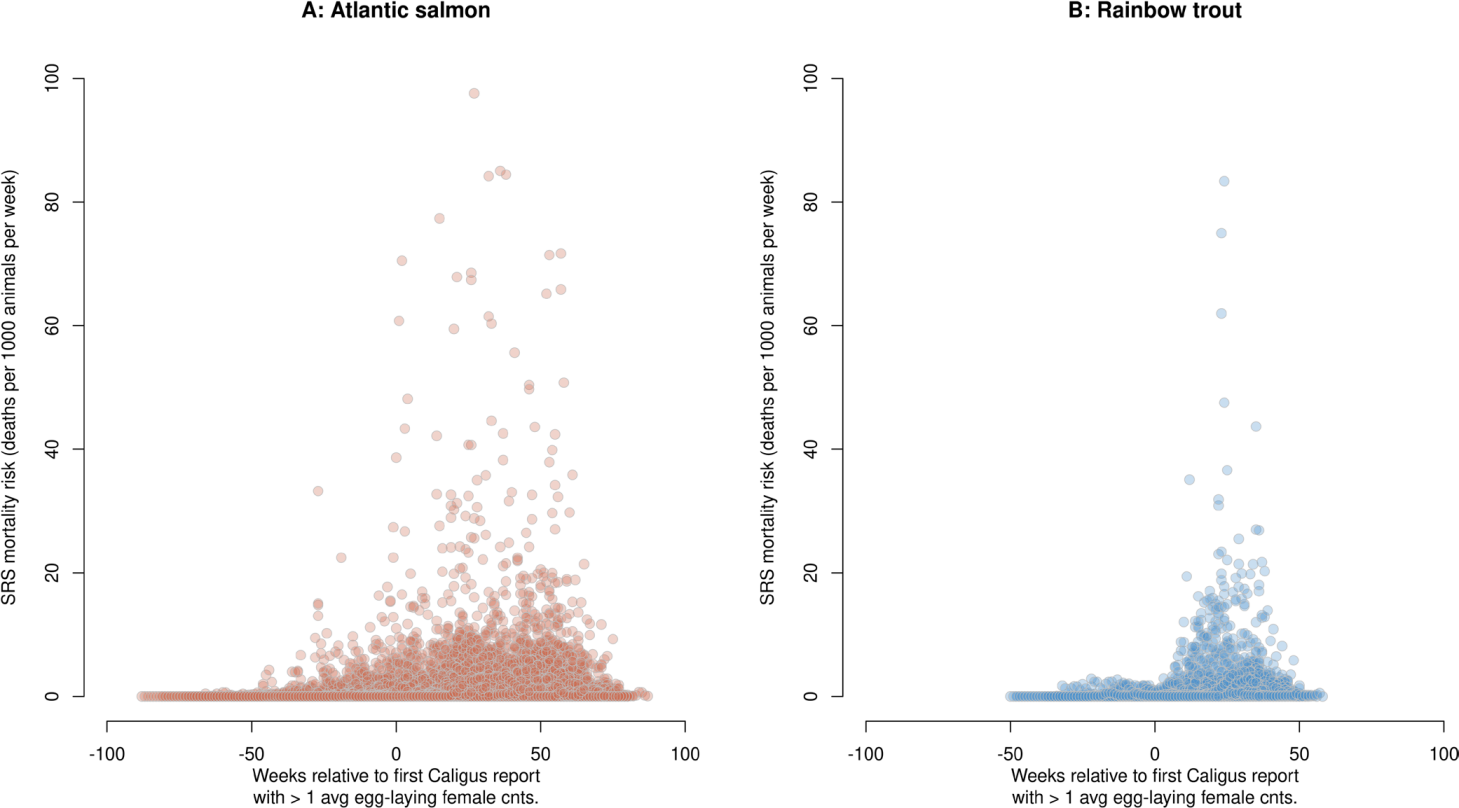
SRS mortality risk relative to the first report of high *Caligus* load (> 1 egg‐laying female per fish) in a production cycle. Each point represents an epidemiological week. Only cycles with at least one high‐load *Caligus* report are included. (A) Atlantic salmon datapoints. (B) Rainbow trout datapoints. No Coho salmon datapoints are included, as none of the production cycles of this species experienced high *Caligus* load reports.

**FIGURE 3:**
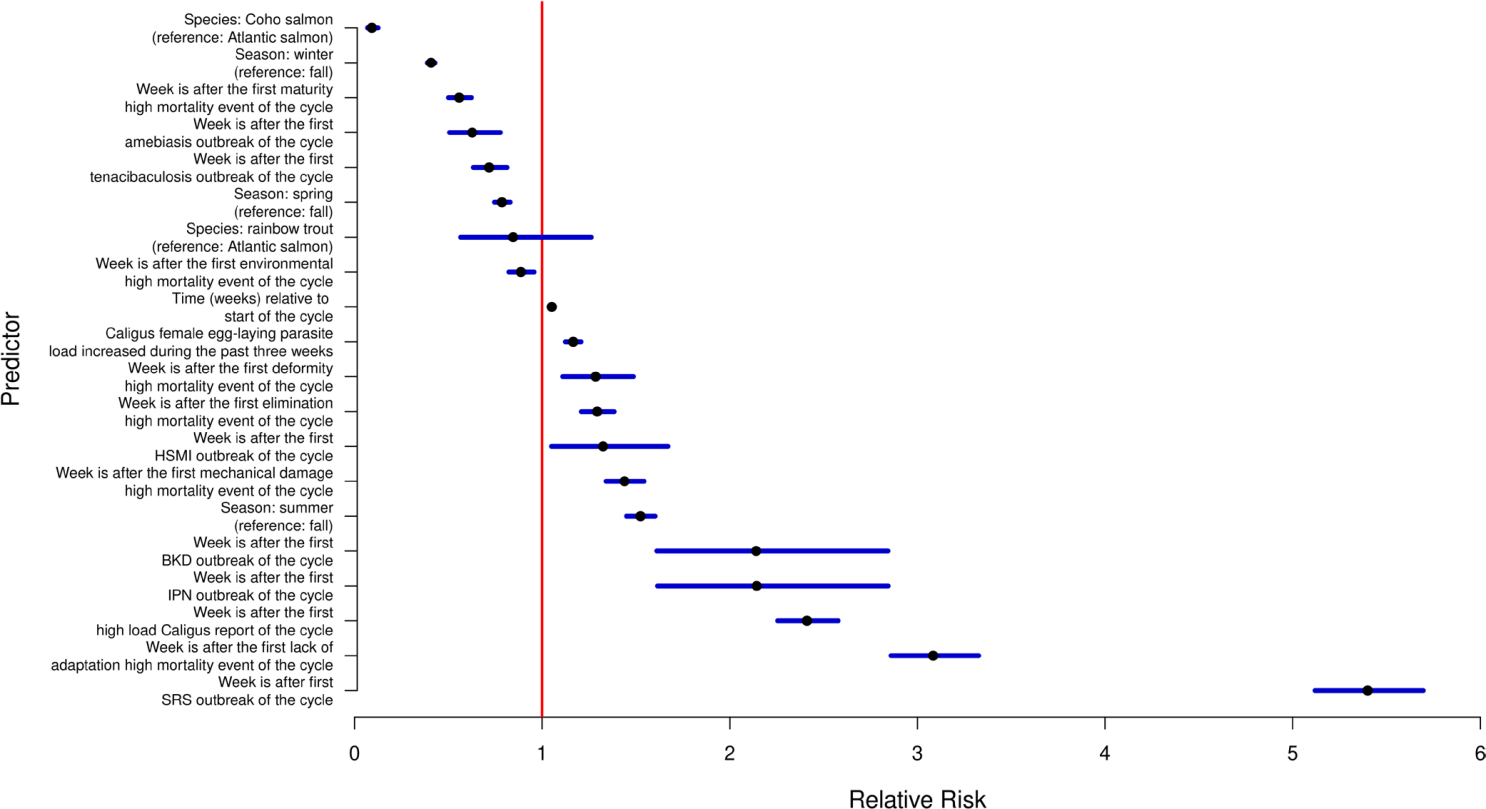
Forest plot of the negative binomial mixed‐effects model for weekly SRS mortality risk (Model 1). AIC values: null model = 728,965.5; full model = 699,391.5; best model = 701,090.

To further investigate the epidemiology of SRS outbreaks and the role of *Caligus* infestations in outbreak dynamics, we explored the association between weekly SRS mortality risk and the timing of the first high‐load *Caligus* report in a production cycle. Visual inspection of the data revealed that SRS mortality risk tends to increase sharply following the first report of a high *Caligus* load within a cycle (Figure 2). This pattern suggests that the first occurrence of a high‐load *Caligus* report may be a strong predictor of increased SRS mortality risk. Accordingly, this variable was included in our generalised model of weekly SRS mortality risk (Model 1; see Figure 3). Consistent with our exploratory analysis, the final model confirmed that the strongest predictor of weekly SRS mortality risk was whether the week occurred after the first SRS outbreak had been declared. This indicates that subsequent weeks following the initial outbreak tend to show higher mortality risk than the initial outbreak period itself. Additionally, Model 1 showed that mortality risk increased progressively over time since the beginning of the production cycle. Importantly, weeks following the first high‐load *Caligus* report had significantly greater SRS mortality risk than weeks preceding it. Moreover, an increase in the *Caligus* infestation rate over the previous 3 weeks was associated with elevated mortality risk, suggesting that rising *Caligus* loads may exacerbate SRS dynamics. The model also controlled for multiple covariates, including other infectious and non‐infectious mortality causes, season, geographical coordinates and salmonid species (Figure 3). Model parameters are summarised in Table S5.

In a consistent fashion with what we found in our visual analysis of the SRS mortality risk relative to the first *Caligus* report of a cycle, our final model (model 1) indicates that the strongest predictor for SRS mortality risk was whether a given week occurred after the first SRS outbreak had been declared, suggesting that subsequent outbreaks result in larger mortality risks than the original outbreak. Model 1 indicates that SRS mortality risk increases continuously as time elapses since the start of the cycle. This model also showed that weeks after the first *Caligus* high load report, the risk had increased compared to previous ones. Another predictor of interest was whether the slope of the average *Caligus* load had increased in the past 3 weeks, indicating that increasing loads are associated with a bigger risk of SRS mortality. In addition to the already discussed predictors, this model controlled for several other causes of mortality, both infectious and non‐infectious, as well as season, geographical coordinates and salmonid species (Figure 3). The model parameters are summarised in Table S5.

### 3.4 Time to the First SRS Outbreak

**FIGURE 4:**
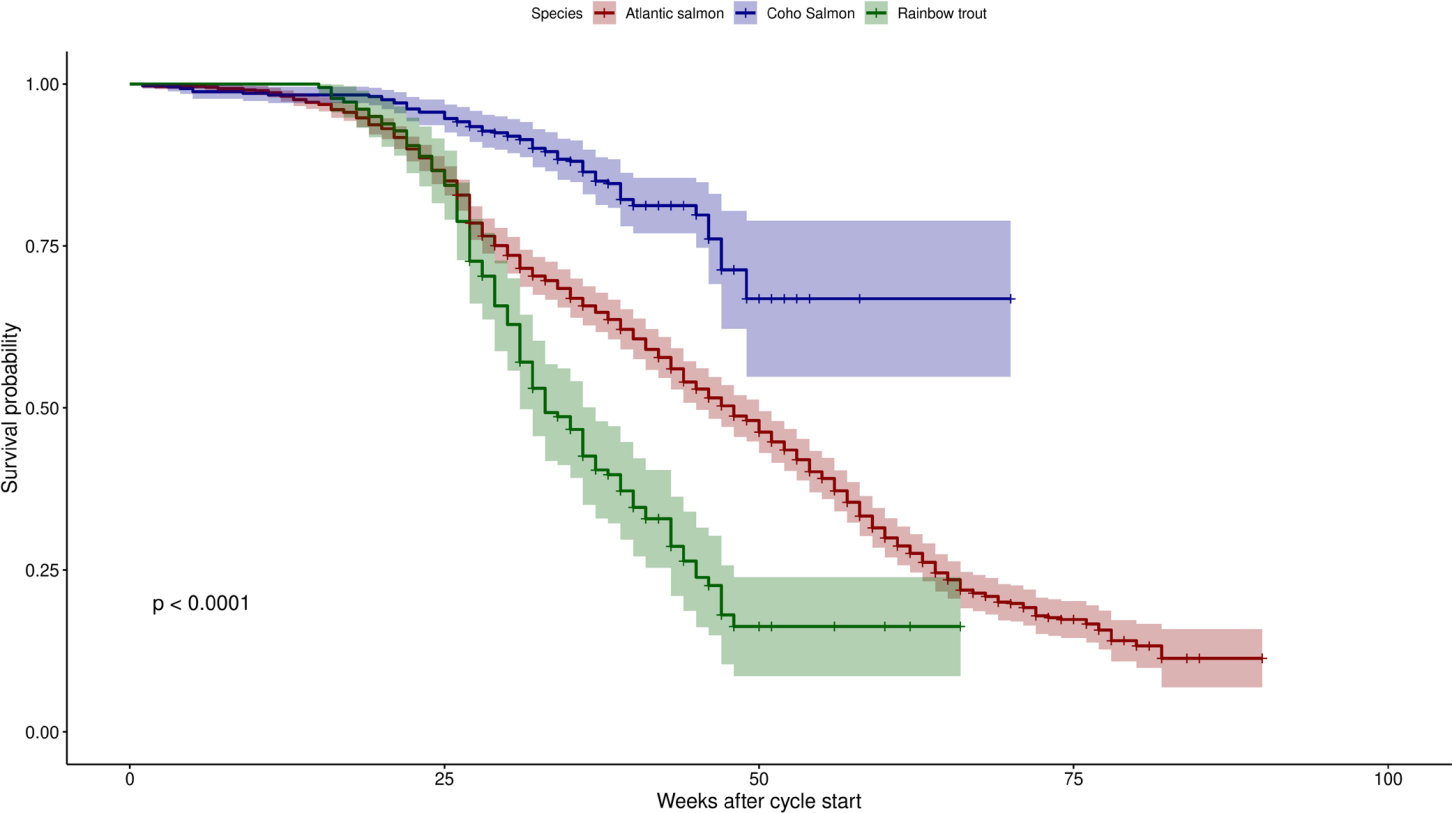
Time to first SRS outbreak in the cycle for each of the three cultured salmonid species. The shown *p*‐value corresponds to the log‐rank test. Shaded areas indicate 95% confidence intervals.

Our previous analyses indicate that the salmonid species is a strong predictor of SRS‐related epidemiological outcomes, so we began our survival analysis with a Kaplan‐Meier estimation of time to the first SRS outbreak, stratified by species (Figure 4). The log‐rank test showed a significant difference in survival distributions across species (*p* < 0.001). Coho salmon cycles exhibited the longest time to outbreak, with fewer than half of the cycles experiencing an SRS outbreak, making the median survival time non‐estimable. In contrast, the median time to the first outbreak was 48 weeks for Atlantic salmon and 33 weeks for rainbow trout, indicating that rainbow trout had the highest hazard of SRS outbreak.

**FIGURE 5:**
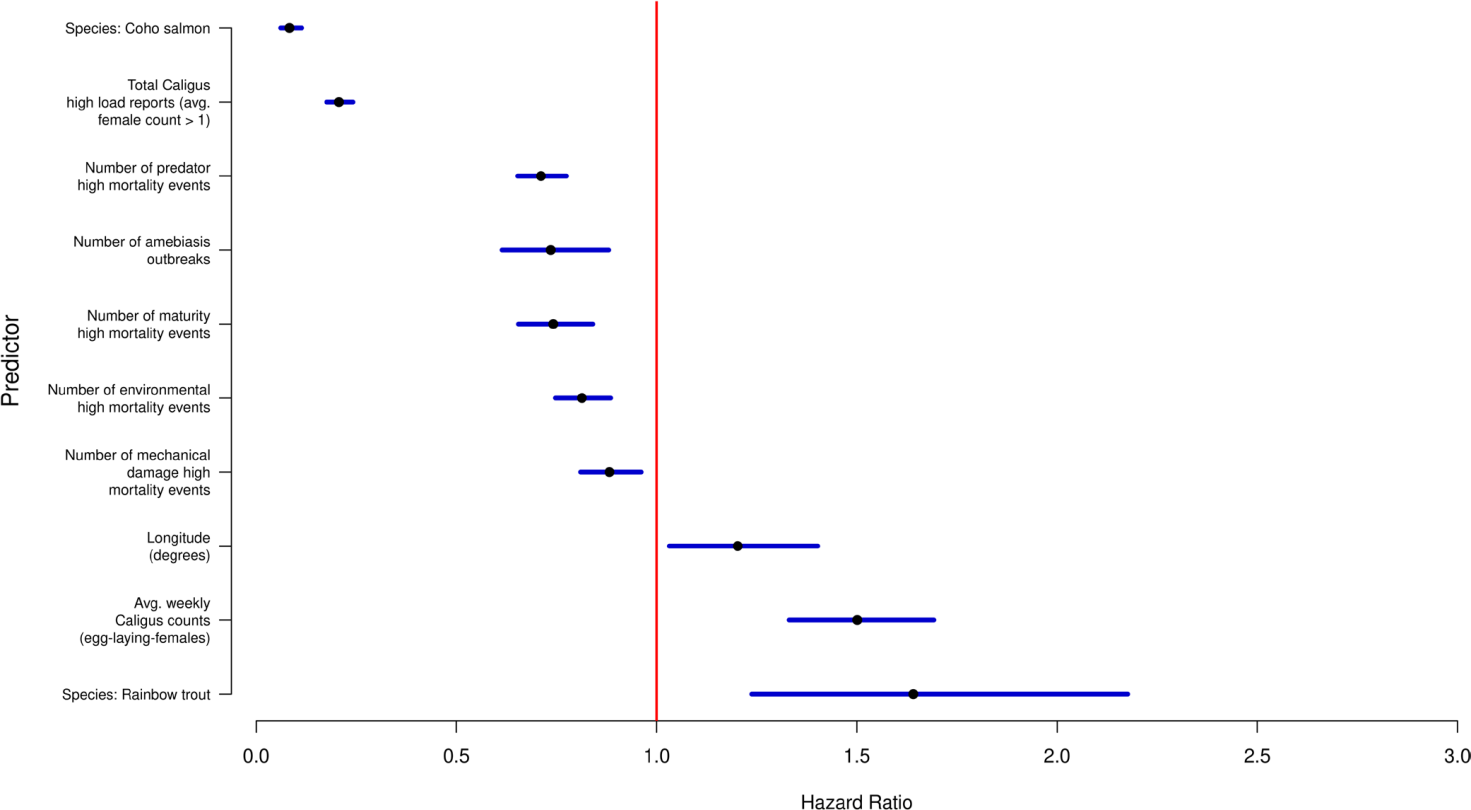
Forest plot for the Cox regression model with frailties of the time to the first SRS outbreak in the salmon production cycle (Model 2). AIC values: null model = 12,083.67; full model = 11,097; final model = 11,089.57.

As expected, the outcome of the Cox model with frailties (model 2) indicated that the hazard of experiencing an SRS outbreak was significantly higher for rainbow trout and significantly lower for coho salmon, relative to Atlantic salmon. On the other hand, the strongest actionable predictor for a shorter time to the first SRS outbreak of a cycle was the average count of female egg‐laying *Caligus* parasites prior to the outbreak (Figure 5). In contrast, a larger number of *Caligus* reports is associated with a lower hazard of reporting the first outbreak of the cycle.

As with model 1, the model adjusted for geographical location, salmonid species and mortality events from other infectious and non‐infectious causes. Notably, a greater number of outbreaks or high‐mortality events from other causes were associated with a reduced hazard of SRS outbreak. The model parameters are summarised in Table S6.

## 4 Discussion

Our time series analysis revealed a notable contrast in trends between SRS mortality risk and *Caligus* infestations over the study period. Specifically, SRS mortality risk remained relatively stable, whereas *Caligus* infestation loads increased significantly. Given the well‐established role of *Caligus* as a physiological stressor and its association with increased vulnerability to infectious diseases (Arriagada et al. 2019; Figueroa et al. 2017; Rozas and Enriquez 2014), this divergence highlights a growing management challenge that warrants reassessment of current lice control strategies. Using our first statistical model (Model 1), we found that SRS mortality risk increased consistently as production cycles progressed. The data supported anecdotal reports from producers suggesting that later phases of the cycle are typically marked by greater SRS burden. Notably, the first high‐load *Caligus* report was a clear inflection point—mortality risk increased significantly in the weeks following this event. A sharp increase was also observed following the onset of the first SRS outbreak, and weeks characterised by an upward trend in *Caligus* loads over the previous 3 weeks exhibited elevated mortality risk. These patterns strongly suggest that both the timing and trajectory of parasite pressure are critical factors in shaping SRS outcomes. From a practical standpoint, these results imply that maintaining low *Caligus* loads and delaying the first SRS outbreak, possibly through early molecular diagnostics and rapid treatment when necessary, should be considered central to effective SRS risk mitigation. Regarding other variables included in our models, most of them represent non‐actionable predictors of risk, such as the season of the year, the salmonid species or the timing of other high‐mortality events, and as such, apart from serving to control for potential confounding, are not proposed as potential ways of risk management in this study.

Our second model (Model 2) assessed factors influencing the timing of the first SRS outbreak in a production cycle. The most actionable predictor for a shorter time to outbreak was the average count of egg‐laying female *Caligus* parasites before the outbreak, reinforcing the conclusion that parasite pressure accelerates SRS onset. Because this is a survival model, the outcome represents the time until the first SRS outbreak in a production cycle; therefore, the identified risk factors should be interpreted as increasing the hazard of the outbreak occurring earlier. Interestingly, we observed that a greater total number of *Caligus* reports during a cycle was associated with a reduced hazard of SRS outbreak. One possible explanation is that more frequent reporting leads to earlier and more consistent implementation of antiparasitic interventions, thus mitigating the buildup of parasite loads and delaying disease onset. However, this interpretation should be treated with caution, as previous work has indicated that sea lice treatments themselves may elevate SRS mortality risk through treatment‐associated stress (González et al. 2016; Meyer et al. 2019). Additionally, the model revealed that high‐mortality events from non‐SRS causes, such as environmental or mechanical factors, were associated with a lower hazard of experiencing an SRS outbreak later in the cycle. This could reflect a survivor bias, whereby the remaining population may be less susceptible to subsequent infections, although this interpretation requires further investigation. Together, these findings underscore the importance of early and sustained parasite control, proactive disease surveillance and timely responses in reducing the burden of SRS in salmon farming.

The results of this study expand upon previous findings, such as those by Arriagada et al. (2019), who reported a link between *Caligus* infestation and cumulative SRS mortality, by demonstrating temporal associations, including how infestation trends influence mortality week‐to‐week and outbreak timing. The observation that subsequent outbreaks yield higher mortality than initial ones is consistent with anecdotal industry reports and highlights the importance of early containment. Estévez et al. (2019) published the results of a structured expert elicitation process where the most important putative risk and protective factors for piscirickettsiosis were identified (Estévez et al. 2019). Several of the risk factors highlighted in that study were also observed here, including seasonal patterns, *Caligus* pressure and prior history of SRS. Regarding seasonality, it is important to note that farming cycles in Chile may begin in any month of the year, and indeed our dataset includes cycles initiated across all calendar months. Nevertheless, as our time‐series analysis demonstrates, SRS infections show a clear seasonal pattern, with significantly higher mortality risk occurring during the warmer months. Based on our current findings, we can offer evidence‐based recommendations to producers and regulatory authorities. First, *Caligus* control emerges as a cornerstone strategy for reducing both SRS mortality risk and the time to outbreak; targeted and timely treatment against sea lice should be prioritised. Second, because subsequent SRS outbreaks are associated with substantially higher mortality risk, proactive efforts to prevent the first outbreak—including improved surveillance, early diagnostics and swift antimicrobial intervention—should be central to farm‐level health management.

While our study offers a comprehensive retrospective analysis of SRS epidemiology in Chilean salmon aquaculture, several limitations must be acknowledged. First, the observational nature of the study design precludes causal inference. Although we identified statistically significant associations between *Caligus* infestation dynamics and both SRS mortality risk and outbreak timing, we cannot confirm that parasite loads cause SRS outbreaks or increased mortality. Confounding variables—such as differences in farm‐level management practices, biosecurity measures, or timing and efficacy of treatments—may influence these associations but were not consistently available in the dataset. Second, our analysis relied on administrative and self‐reported data collected through national surveillance systems. Although Sernapesca mandates standardised reporting, potential inconsistencies or delays in reporting, differences in diagnostic confirmation or underreporting—especially of subclinical infections—may introduce reporting bias. Furthermore, the precise timing of antiparasitic and antimicrobial treatments was not included, limiting our ability to account for potential interventions that could alter outbreak risk or duration. Third, the observed association between non‐SRS high‐mortality events and a reduced hazard of subsequent SRS outbreaks raises interesting biological hypotheses—such as survival bias or changes in fish susceptibility—but we lack individual‐level data (e.g., genetic resistance, immune parameters) or pathogen strain information to explore these mechanisms further. Additionally, while we controlled for salmonid species, seasonality and geography, we recognise that environmental variables such as water temperature, salinity and hydrodynamics—previously shown to influence both *Caligus* and SRS dynamics—were not available at the farm‐week level with sufficient resolution for inclusion in our models. Fourth, as production cycles are not explicitly indicated in the SIFA database, we had to infer start and end dates by identifying time gaps in reporting, as described in the Methods section. This approach carries the inherent risk of misidentifying some cycles, and it could be argued that the shorter cycles in our dataset may correspond to minimal‐operation periods in between proper production cycles, the nature of which could differ systematically from longer cycles. Without explicit indications for each cycle's duration, our ability to identify individual production cycles is at best an approximation. Finally, while we standardised numeric variables and used mixed‐effects models to address clustering and hierarchical data structures, model assumptions (e.g., proportional hazards in Cox regression, overdispersion in negative binomial models) could still influence results if not fully met across all strata. Despite these limitations, the size and temporal scope of our dataset, combined with robust modelling approaches, support the reliability of our findings. Nonetheless, future studies should aim to incorporate prospective designs, more granular environmental and treatment data and mechanistic insights to validate and extend these findings.

In summary, the present work comprised a retrospective epidemiological study of the entire Chilean salmoniculture system between the years 2014 and 2021. The insights gathered herein enhance our understanding of the factors that influence SRS mortality risk and the timing of outbreaks, with *Caligus* infestation control and SRS outbreak prevention emerging as central, actionable drivers of disease dynamics. These findings can support regulators and producers in refining health management strategies with a sharper focus on early warning and intervention. Further studies should aim to enrich these findings by evaluating additional predictors—particularly considering evolving management practices, regulatory frameworks and climate‐related changes. Ideally, prospective cohort studies should be designed to test causal hypotheses, explore the effectiveness of targeted interventions and assess whether early *Caligus* monitoring and treatment strategies can consistently delay or prevent SRS outbreaks. Additionally, future work should investigate the interactions between SRS and other mortality causes, both infectious and non‐infectious, to determine whether early‐life events or co‐infections modulate disease susceptibility later in the production cycle. Incorporating environmental and genetic data, improving temporal resolution of management actions (e.g., treatment timing) and integrating novel molecular diagnostics will be essential to building predictive tools and adaptive management frameworks that are responsive to real‐time conditions. These efforts will help ensure the long‐term sustainability and resilience of the Chilean salmon farming industry.

## 5 Conclusions

This study provides a comprehensive epidemiological assessment of SRS in Chilean salmon farming and highlights the pivotal role of *Caligus* infestation dynamics. Maintaining low *Caligus* loads and delaying the first SRS outbreak through early detection and intervention of this bacterial disease could reduce both mortality and antimicrobial use. These findings offer practical, actionable insights for producers and regulators aiming to improve disease management in the Chilean salmon farming system.

## Author Contributions

**Benjamín Diethelm‐Varela:** conceptualisation, investigation, methodology, visualisation, writing – original draft. **Nicolhole Atero:** writing – review and editing, investigation. **Francisca Córdova‐Bührle:** writing – review and editing. **Enrico L. Rezende:** supervision, writing – review and editing, methodology. **Stefan Gelcich:** supervision, writing – review and editing. **Osvaldo Sandoval:** writing – review and editing, investigation. **Carlos Navarro:** writing – review and editing, investigation. **Fernando O. Mardones:** supervision, writing – review and editing, investigation, project administration and funding acquisition.

## Funding

B.D.‐V. is a beneficiary of the ANID National Doctoral Scholarship #21221163. This research project was funded by the FONDECYT Regular Project #1191675. S.G. received support from the ANID‐Millennium Science Initiative Program—code ICN 2019_015.

## Ethics Statement

In accordance with institutional and national guidelines, ethical approval was not required for this research.

## Conflicts of Interest

The authors declare no conflicts of interest.

## Supporting information


**Figure S1:** Time series of mortality risk for various infectious diseases in the Chilean salmoniculture system in the Los Lagos and Aysén regions between the years 2014 and 2021. Each datapoint corresponds to an epidemiological week within a production cycle.
**Figure S2:** Time series of mortality risk for various non‐infectious causes in the Chilean salmoniculture system in the Los Lagos and Aysén regions between the years 2014 and 2021. SRS mortalities are plotted too for reference. Each datapoint corresponds to an epidemiological week within a production cycle.
**Figure S3:** Decomposition of the time series of the SRS mortality risk over time.
**Table S1:** Coefficients, standard errors, statistics, *p*‐values and confidence intervals for the mixed effects linear model to assess the seasonality of the SRS mortality risk over time. Conditional *R* ^2^: 0.76.
**Table S2:** Coefficients, standard errors, statistics, *p*‐values and confidence intervals for the mixed effects linear model to assess the trend of the SRS mortality risk over time. Conditional *R* ^2^: 0.44.
**Figure S4:** Decomposition of the time series of the *Caligus* infestation load over time.
**Table S3:** Coefficients, standard errors, statistics, *p*‐values and confidence intervals for the mixed effects linear model to assess the seasonality of the *Caligus* infestation load over time. Conditional *R* ^2^: 0.88.
**Table S4:** Coefficients, standard errors, statistics, *p*‐values and confidence intervals for the mixed effects linear model to assess the trend of the *Caligus* infestation load over time. Conditional *R* ^2^: 0.8.
**Table S5:** Coefficients, standard errors, statistics, *p*‐values and confidence intervals for the mixed effects negative binomial model that explains the SRS weekly mortality risk (Model 1).
**Table S6:** Coefficients, standard errors, statistics, *p*‐values and confidence intervals for the Cox regression model with frailties that explains the time to the first SRS outbreak in a production cycle (Model 2).

## Data Availability

Epidemiological datasets and all analysis scripts used in this study will be made available upon reasonable request.
