# Supplementary material for: Epidemiology of Salmonid Rickettsial Septicemia (SRS) in Farmed Salmon: The Role of Sea Lice Infestations in Mortality Risk
Source: J Fish Dis. 2025 Dec 9;49(5):e70097. doi: 10.1111/jfd.70097 (PMC13051326; doi:10.1111/jfd.70097)
Supplement: Supplementary file 1 — Figure S1: Time series of mortality risk for various infectious diseases in the Chilean salmoniculture system in the Los Lagos and Aysén regions between the years 2014 and 2021. Each datapoint corresponds to an epidemiological week within a production cycle. Figure S2: Time series of mortality risk for various non‐infectious causes in the Chilean salmoniculture system in the Los Lagos and Aysén regions between the years 2014 and 2021. SRS mortalities are plotted too for reference. Each datapoint corresponds to an epidemiological week within a production cycle. Figure S3: Decomposition of the time series of the SRS mortality risk over time. Table S1: Coefficients, standard errors, statistics, p‐values and confidence intervals for the mixed effects linear model to assess the seasonality of the SRS mortality risk over time. Conditional R 2: 0.76. Table S2: Coefficients, standard errors, statistics, p‐values and confidence intervals for the mixed effects linear model to assess the trend of the SRS mortality risk over time. Conditional R 2: 0.44. Figure S4: Decomposition of the time series of the Caligus infestation load over time. Table S3: Coefficients, standard errors, statistics, p‐values and confidence intervals for the mixed effects linear model to assess the seasonality of the Caligus infestation load over time. Conditional R 2: 0.88. Table S4: Coefficients, standard errors, statistics, p‐values and confidence intervals for the mixed effects linear model to assess the trend of the Caligus infestation load over time. Conditional R 2: 0.8. Table S5: Coefficients, standard errors, statistics, p‐values and confidence intervals for the mixed effects negative binomial model that explains the SRS weekly mortality risk (Model 1). Table S6: Coefficients, standard errors, statistics, p‐values and confidence intervals for the Cox regression model with frailties that explains the time to the first SRS outbreak in a production cycle (Model 2). [file JFD-49-e70097-s001.docx]

# Supplementary Information

| 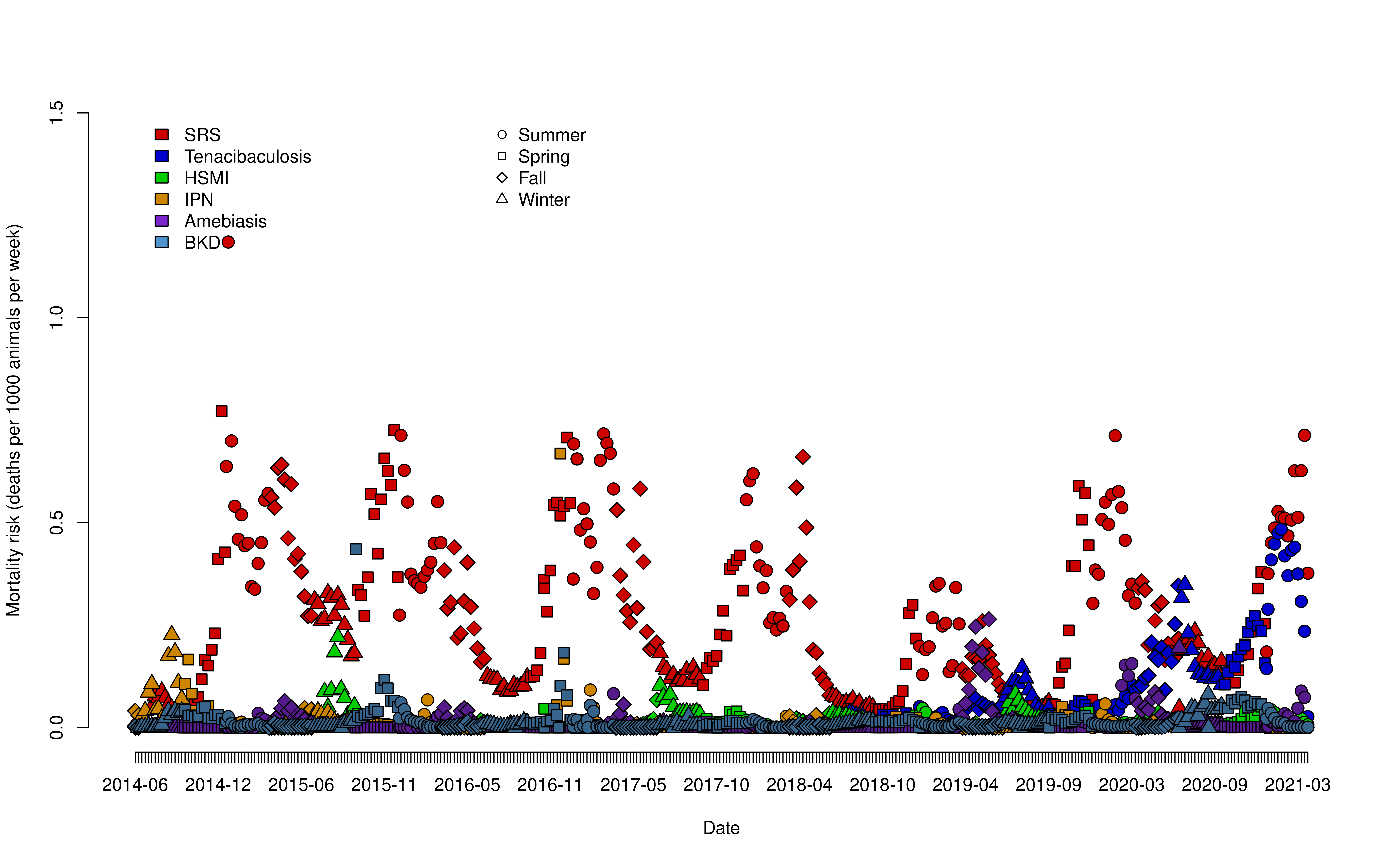 Figure S1: Time series of mortality risk for various infectious diseases in the Chilean salmoniculture system in the Los Lagos and Aysén regions between the 2014-2021 period. Each datapoint corresponds to an epidemiological week within a production cycle. |
| --- |

| 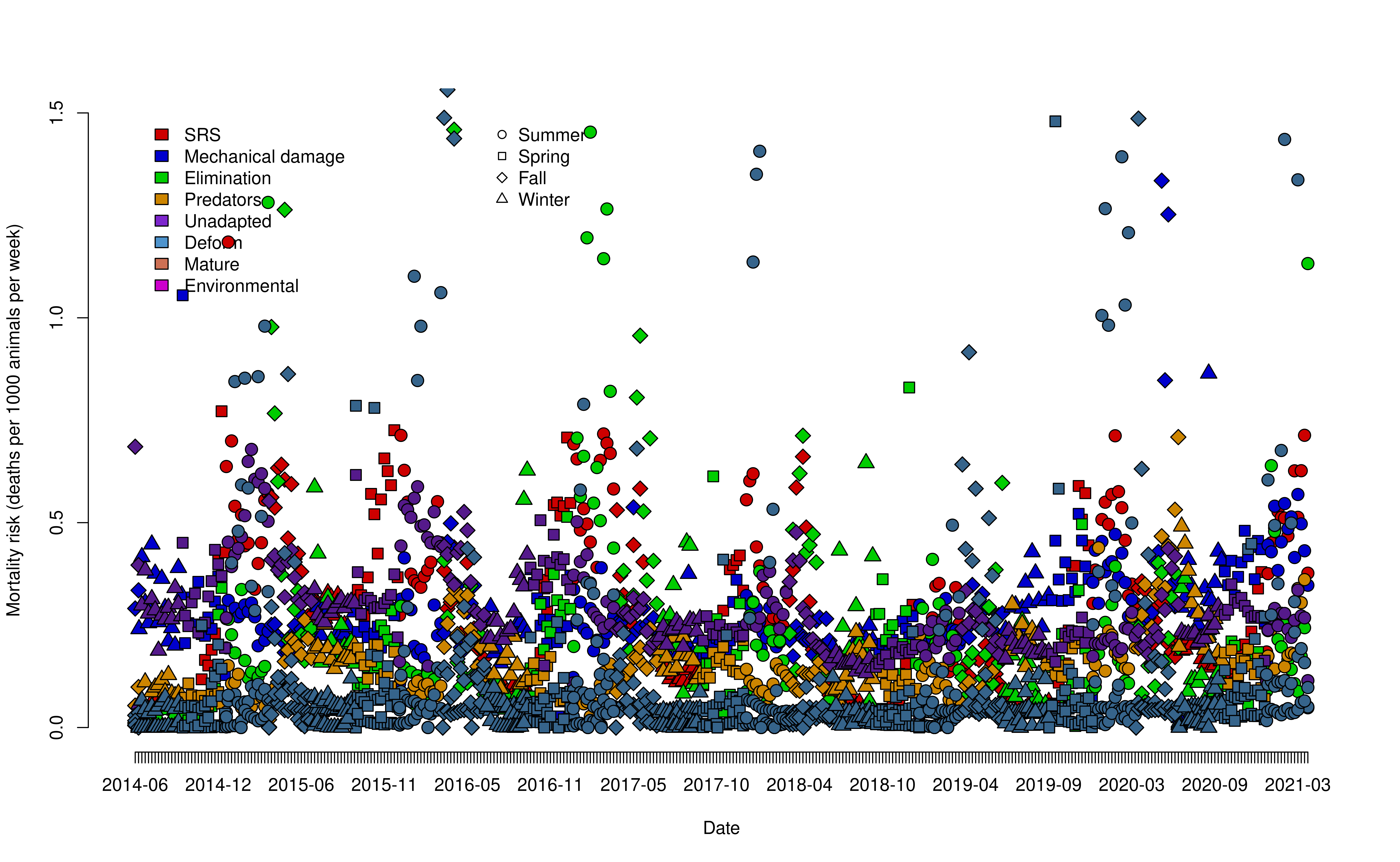 Figure S2: Time series of mortality risk for various non-infectious causes in the Chilean salmoniculture system in the Los Lagos and Aysén regions between the 2014-2021 period. SRS mortalities are plotted too for reference. Each datapoint corresponds to an epidemiological week within a production cycle |
| --- |

| 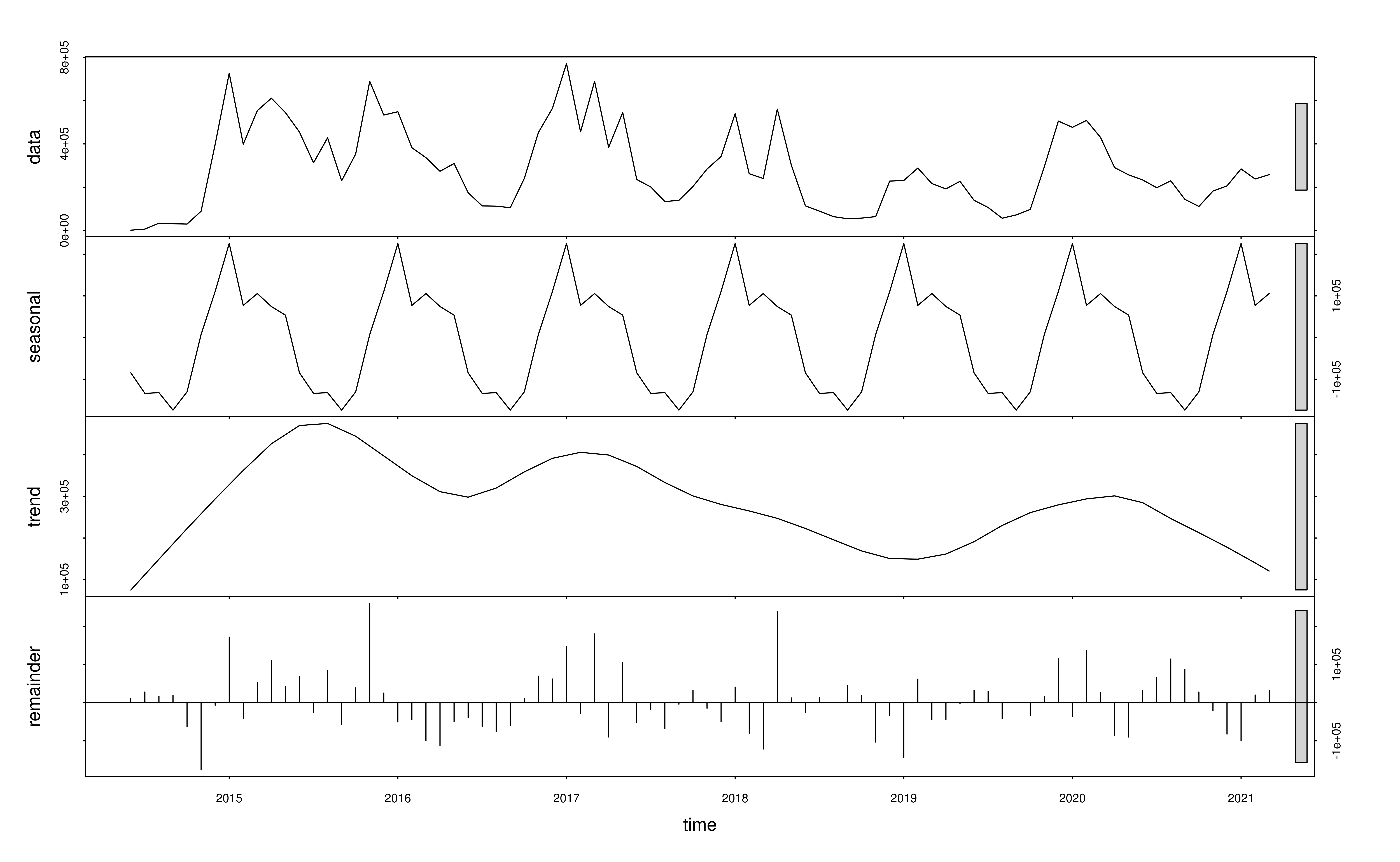 Figure S3: Decomposition of the time series of the SRS mortality risk over time |
| --- |

| Table S1: Coefficients, standard errors, statistics, p-values, and confidence intervals for the mixed effects linear model to assess the seasonality of the SRS mortality risk over time. Conditional R^2^: 0.76   \| **Effect** \| **Group** \| **Term** \| **Estimate** \| **Standard error** \| **Statistic** \| **df** \| **P-value** \| **Lower CI95%** \| **Upper CI95%** \| \| --- \| --- \| --- \| --- \| --- \| --- \| --- \| --- \| --- \| --- \| \| fixed \| NA \| (Intercept) \| 0.46 \| 0.05 \| 10.15 \| 24.31 \| 0 \| 0.36 \| 0.55 \| \| fixed \| NA \| Month 2 \| -0.05 \| 0.05 \| -1.03 \| 63.13 \| 0.31 \| -0.14 \| 0.05 \| \| fixed \| NA \| Month 3 \| -0.03 \| 0.05 \| -0.64 \| 63.13 \| 0.53 \| -0.12 \| 0.06 \| \| fixed \| NA \| Month 4 \| -0.09 \| 0.05 \| -1.79 \| 63.79 \| 0.08 \| -0.19 \| 0.01 \| \| fixed \| NA \| Month 5 \| -0.14 \| 0.05 \| -2.76 \| 63.79 \| 0.01 \| -0.23 \| -0.04 \| \| fixed \| NA \| Month 6 \| -0.28 \| 0.05 \| -6.02 \| 64.07 \| 0 \| -0.38 \| -0.19 \| \| fixed \| NA \| Month 7 \| -0.32 \| 0.05 \| -6.78 \| 64.07 \| 0 \| -0.42 \| -0.23 \| \| fixed \| NA \| Month 8 \| -0.32 \| 0.05 \| -6.8 \| 64.07 \| 0 \| -0.42 \| -0.23 \| \| fixed \| NA \| Month 9 \| -0.35 \| 0.05 \| -7.3 \| 64.07 \| 0 \| -0.44 \| -0.25 \| \| fixed \| NA \| Month 10 \| -0.31 \| 0.05 \| -6.6 \| 64.07 \| 0 \| -0.41 \| -0.22 \| \| fixed \| NA \| Month 11 \| -0.17 \| 0.05 \| -3.68 \| 64.07 \| 0 \| -0.27 \| -0.08 \| \| fixed \| NA \| Month 12 \| -0.03 \| 0.05 \| -0.68 \| 64.07 \| 0.5 \| -0.13 \| 0.06 \| \| random \| year \| sd__(Intercept) \| 0.09 \| NA \| NA \| NA \| NA \| NA \| NA \| \| random \| Residual \| sd__Observation \| 0.09 \| NA \| NA \| NA \| NA \| NA \| NA \| |
| --- | --- | --- | --- | --- | --- | --- | --- | --- | --- | --- | --- | --- | --- | --- | --- | --- | --- | --- | --- | --- | --- | --- | --- | --- | --- | --- | --- | --- | --- | --- | --- | --- | --- | --- | --- | --- | --- | --- | --- | --- | --- | --- | --- | --- | --- | --- | --- | --- | --- | --- | --- | --- | --- | --- | --- | --- | --- | --- | --- | --- | --- | --- | --- | --- | --- | --- | --- | --- | --- | --- | --- | --- | --- | --- | --- | --- | --- | --- | --- | --- | --- | --- | --- | --- | --- | --- | --- | --- | --- | --- | --- | --- | --- | --- | --- | --- | --- | --- | --- | --- | --- | --- | --- | --- | --- | --- | --- | --- | --- | --- | --- | --- | --- | --- | --- | --- | --- | --- | --- | --- | --- | --- | --- | --- | --- | --- | --- | --- | --- | --- | --- | --- | --- | --- | --- | --- | --- | --- | --- | --- | --- | --- | --- | --- | --- | --- | --- | --- | --- | --- |

| Table S2: Coefficients, standard errors, statistics, p-values, and confidence intervals for the mixed effects linear model to assess the trend of the SRS mortality risk over time. Conditional R^2^: 0.44   \| **Effect** \| **Group** \| **Term** \| **Estimate** \| **Standard error** \| **Statistic** \| **df** \| **P-value** \| **Lower CI95%** \| **Upper CI95%** \| \| --- \| --- \| --- \| --- \| --- \| --- \| --- \| --- \| --- \| --- \| \| fixed \| NA \| (Intercept) \| 0.34 \| 0.09 \| 3.7 \| 4.47 \| 0.02 \| 0.1 \| 0.58 \| \| fixed \| NA \| index \| 0 \| 0 \| -0.67 \| 5.24 \| 0.53 \| -0.01 \| 0 \| \| random \| year \| sd__(Intercept) \| 0.13 \| NA \| NA \| NA \| NA \| NA \| NA \| \| random \| Residual \| sd__Observation \| 0.15 \| NA \| NA \| NA \| NA \| NA \| NA \| |
| --- | --- | --- | --- | --- | --- | --- | --- | --- | --- | --- | --- | --- | --- | --- | --- | --- | --- | --- | --- | --- | --- | --- | --- | --- | --- | --- | --- | --- | --- | --- | --- | --- | --- | --- | --- | --- | --- | --- | --- | --- | --- | --- | --- | --- | --- | --- | --- | --- | --- | --- |

| 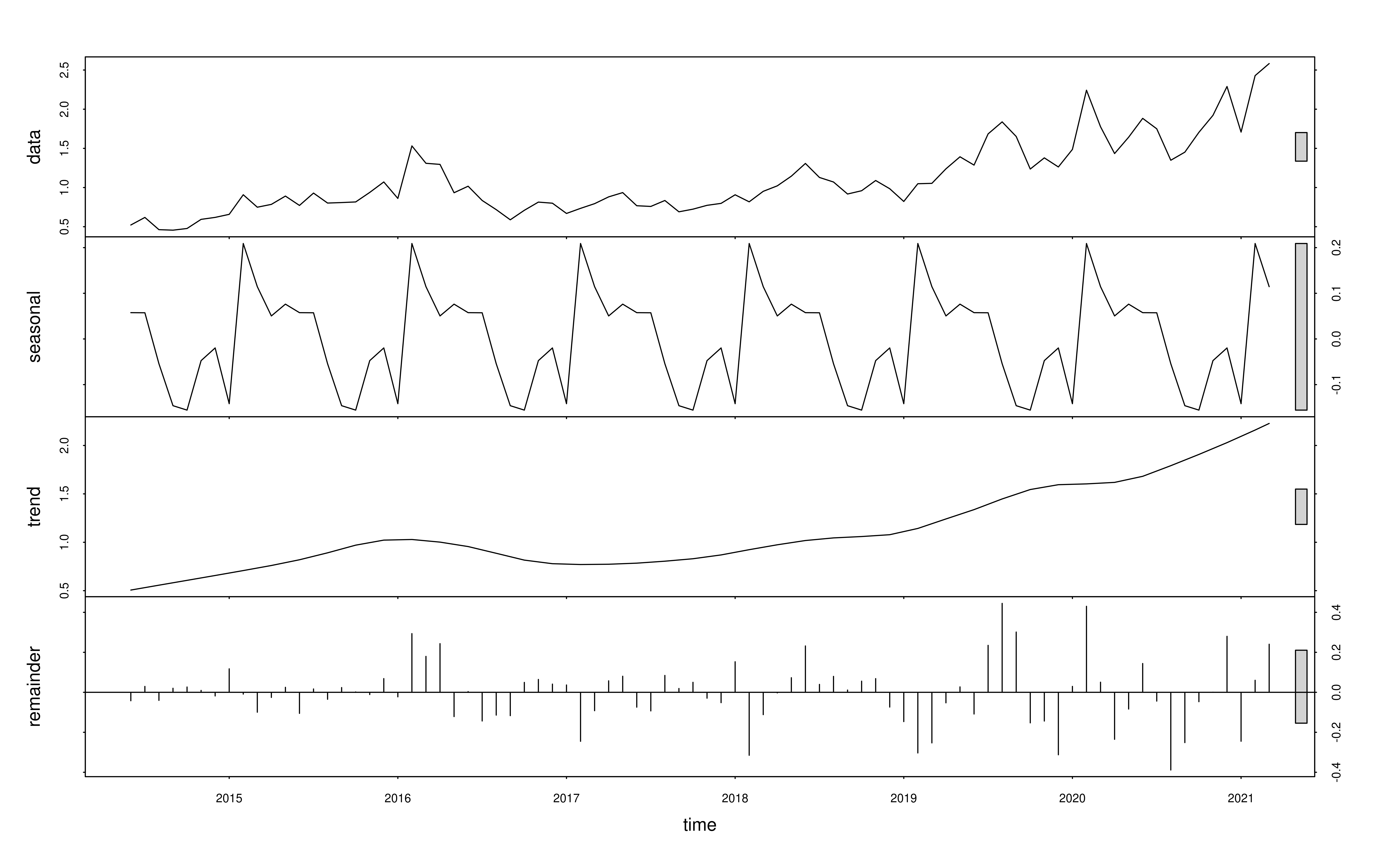 Figure S4: Decomposition of the time series of the *Caligus* infestation load over time |
| --- |

| Table S3: Coefficients, standard errors, statistics, p-values, and confidence intervals for the mixed effects linear model to assess the seasonality of the *Caligus* infestation load over time. Conditional R^2^: 0.88.   \| **Effect** \| **Group** \| **Term** \| **Estimate** \| **Standard error** \| **Statistic** \| **df** \| **P-value** \| **Lower CI95%** \| **Upper CI95%** \| \| --- \| --- \| --- \| --- \| --- \| --- \| --- \| --- \| --- \| --- \| \| fixed \| NA \| (Intercept) \| 0.92 \| 0.21 \| 4.31 \| 8.9 \| 0 \| 0.44 \| 1.41 \| \| fixed \| NA \| Month 2 \| 0.37 \| 0.11 \| 3.24 \| 62.75 \| 0 \| 0.14 \| 0.6 \| \| fixed \| NA \| Month 3 \| 0.3 \| 0.11 \| 2.63 \| 62.75 \| 0.01 \| 0.07 \| 0.53 \| \| fixed \| NA \| Month 4 \| 0.25 \| 0.12 \| 2.09 \| 62.9 \| 0.04 \| 0.01 \| 0.49 \| \| fixed \| NA \| Month 5 \| 0.3 \| 0.12 \| 2.48 \| 62.9 \| 0.02 \| 0.06 \| 0.54 \| \| fixed \| NA \| Month 6 \| 0.3 \| 0.12 \| 2.6 \| 62.96 \| 0.01 \| 0.07 \| 0.54 \| \| fixed \| NA \| Month 7 \| 0.32 \| 0.12 \| 2.78 \| 62.96 \| 0.01 \| 0.09 \| 0.56 \| \| fixed \| NA \| Month 8 \| 0.23 \| 0.12 \| 2.01 \| 62.96 \| 0.05 \| 0 \| 0.47 \| \| fixed \| NA \| Month 9 \| 0.16 \| 0.12 \| 1.38 \| 62.96 \| 0.17 \| -0.07 \| 0.39 \| \| fixed \| NA \| Month 10 \| 0.17 \| 0.12 \| 1.46 \| 62.96 \| 0.15 \| -0.06 \| 0.4 \| \| fixed \| NA \| Month 11 \| 0.3 \| 0.12 \| 2.54 \| 62.96 \| 0.01 \| 0.06 \| 0.53 \| \| fixed \| NA \| Month 12 \| 0.34 \| 0.12 \| 2.93 \| 62.96 \| 0 \| 0.11 \| 0.57 \| \| random \| year \| sd__(Intercept) \| 0.56 \| NA \| NA \| NA \| NA \| NA \| NA \| \| random \| Residual \| sd__Observation \| 0.21 \| NA \| NA \| NA \| NA \| NA \| NA \| |
| --- | --- | --- | --- | --- | --- | --- | --- | --- | --- | --- | --- | --- | --- | --- | --- | --- | --- | --- | --- | --- | --- | --- | --- | --- | --- | --- | --- | --- | --- | --- | --- | --- | --- | --- | --- | --- | --- | --- | --- | --- | --- | --- | --- | --- | --- | --- | --- | --- | --- | --- | --- | --- | --- | --- | --- | --- | --- | --- | --- | --- | --- | --- | --- | --- | --- | --- | --- | --- | --- | --- | --- | --- | --- | --- | --- | --- | --- | --- | --- | --- | --- | --- | --- | --- | --- | --- | --- | --- | --- | --- | --- | --- | --- | --- | --- | --- | --- | --- | --- | --- | --- | --- | --- | --- | --- | --- | --- | --- | --- | --- | --- | --- | --- | --- | --- | --- | --- | --- | --- | --- | --- | --- | --- | --- | --- | --- | --- | --- | --- | --- | --- | --- | --- | --- | --- | --- | --- | --- | --- | --- | --- | --- | --- | --- | --- | --- | --- | --- | --- | --- |

| Table S4: Coefficients, standard errors, statistics, p-values, and confidence intervals for the mixed effects linear model to assess the trend of the *Caligus* infestation load over time. Conditional R^2^: 0.8.   \| **Effect** \| **Group** \| **Term** \| **Estimate** \| **Standard error** \| **Statistic** \| **df** \| **P-value** \| **Lower CI95%** \| **Upper CI95%** \| \| --- \| --- \| --- \| --- \| --- \| --- \| --- \| --- \| --- \| --- \| \| fixed \| NA \| (Intercept) \| 0.48 \| 0.16 \| 3.05 \| 6.84 \| 0.02 \| 0.11 \| 0.85 \| \| fixed \| NA \| index \| 0.02 \| 0 \| 5.21 \| 8.16 \| 0 \| 0.01 \| 0.02 \| \| random \| year \| sd__(Intercept) \| 0.23 \| NA \| NA \| NA \| NA \| NA \| NA \| \| random \| Residual \| sd__Observation \| 0.23 \| NA \| NA \| NA \| NA \| NA \| NA \| |
| --- | --- | --- | --- | --- | --- | --- | --- | --- | --- | --- | --- | --- | --- | --- | --- | --- | --- | --- | --- | --- | --- | --- | --- | --- | --- | --- | --- | --- | --- | --- | --- | --- | --- | --- | --- | --- | --- | --- | --- | --- | --- | --- | --- | --- | --- | --- | --- | --- | --- | --- |

| Table S5: Coefficients, standard errors, statistics, p-values, and confidence intervals for the mixed effects negative binomial model that explains the SRS weekly mortality risk (Model 1)   \| **Effect** \| **Group** \| **Term** \| **Estimate** \| **Standard error** \| **Statistic** \| **P-value** \| **Lower CI95%** \| **Upper CI95%** \| \| --- \| --- \| --- \| --- \| --- \| --- \| --- \| --- \| --- \| \| fixed \| NA \| (Intercept) \| -13.08 \| 0.1 \| -131.39 \| 0 \| -13.27 \| -12.88 \| \| fixed \| NA \| time \| 0.05 \| 0 \| 51.88 \| 0 \| 0.05 \| 0.05 \| \| fixed \| NA \| Species: Coho \| -2.38 \| 0.15 \| -15.54 \| 0 \| -2.68 \| -2.08 \| \| fixed \| NA \| Species: Rainbow trout \| -0.17 \| 0.2 \| -0.82 \| 0.41 \| -0.57 \| 0.23 \| \| fixed \| NA \| Season: spring \| -0.24 \| 0.03 \| -8.87 \| 0 \| -0.29 \| -0.19 \| \| fixed \| NA \| Season: summer \| 0.42 \| 0.03 \| 16.51 \| 0 \| 0.37 \| 0.47 \| \| fixed \| NA \| Season: winter \| -0.9 \| 0.03 \| -34.6 \| 0 \| -0.95 \| -0.85 \| \| fixed \| NA \| Week after first environmental high mortality event \| -0.12 \| 0.04 \| -3.13 \| 0 \| -0.2 \| -0.04 \| \| fixed \| NA \| Week after first elimination high mortality event \| 0.26 \| 0.03 \| 7.42 \| 0 \| 0.19 \| 0.32 \| \| fixed \| NA \| Week after first mechanical damage high mortality event \| 0.36 \| 0.04 \| 10.16 \| 0 \| 0.29 \| 0.43 \| \| fixed \| NA \| Week after first lack of adaptation high mortality event \| 1.13 \| 0.04 \| 29.2 \| 0 \| 1.05 \| 1.2 \| \| fixed \| NA \| Week after first deformity high mortality event \| 0.25 \| 0.07 \| 3.37 \| 0 \| 0.1 \| 0.4 \| \| fixed \| NA \| Week after first maturity high mortality event \| -0.58 \| 0.06 \| -10.45 \| 0 \| -0.69 \| -0.47 \| \| fixed \| NA \| Week after first IPN outbreak \| 0.76 \| 0.14 \| 5.28 \| 0 \| 0.48 \| 1.05 \| \| fixed \| NA \| Week after first amebiasis outbreak \| -0.47 \| 0.11 \| -4.25 \| 0 \| -0.68 \| -0.25 \| \| fixed \| NA \| Week after first HSMI outbreak \| 0.28 \| 0.12 \| 2.37 \| 0.02 \| 0.05 \| 0.51 \| \| fixed \| NA \| Week after first tenacibaculosis outbreak \| -0.33 \| 0.06 \| -5.22 \| 0 \| -0.46 \| -0.21 \| \| fixed \| NA \| Week after first BKD outbreak \| 0.76 \| 0.14 \| 5.25 \| 0 \| 0.48 \| 1.04 \| \| fixed \| NA \| Caligus load increased in last three weeks \| 0.15 \| 0.02 \| 8.4 \| 0 \| 0.12 \| 0.19 \| \| fixed \| NA \| Week is after first Caligus report of the cycle \| 0.88 \| 0.03 \| 25.87 \| 0 \| 0.81 \| 0.95 \| \| fixed \| NA \| Week is after first SRS outbreak of the cycle \| 1.69 \| 0.03 \| 61.79 \| 0 \| 1.63 \| 1.74 \| \| random \| cycle:farm:acs \| sd__(Intercept) \| 1.98 \| NA \| NA \| NA \| 0.21 \| 0.22 \| \| random \| farm:acs \| sd__(Intercept) \| 0.83 \| NA \| NA \| NA \| NA \| NA \| \| random \| acs \| sd__(Intercept) \| 0.37 \| NA \| NA \| NA \| NA \| NA \| |
| --- | --- | --- | --- | --- | --- | --- | --- | --- | --- | --- | --- | --- | --- | --- | --- | --- | --- | --- | --- | --- | --- | --- | --- | --- | --- | --- | --- | --- | --- | --- | --- | --- | --- | --- | --- | --- | --- | --- | --- | --- | --- | --- | --- | --- | --- | --- | --- | --- | --- | --- | --- | --- | --- | --- | --- | --- | --- | --- | --- | --- | --- | --- | --- | --- | --- | --- | --- | --- | --- | --- | --- | --- | --- | --- | --- | --- | --- | --- | --- | --- | --- | --- | --- | --- | --- | --- | --- | --- | --- | --- | --- | --- | --- | --- | --- | --- | --- | --- | --- | --- | --- | --- | --- | --- | --- | --- | --- | --- | --- | --- | --- | --- | --- | --- | --- | --- | --- | --- | --- | --- | --- | --- | --- | --- | --- | --- | --- | --- | --- | --- | --- | --- | --- | --- | --- | --- | --- | --- | --- | --- | --- | --- | --- | --- | --- | --- | --- | --- | --- | --- | --- | --- | --- | --- | --- | --- | --- | --- | --- | --- | --- | --- | --- | --- | --- | --- | --- | --- | --- | --- | --- | --- | --- | --- | --- | --- | --- | --- | --- | --- | --- | --- | --- | --- | --- | --- | --- | --- | --- | --- | --- | --- | --- | --- | --- | --- | --- | --- | --- | --- | --- | --- | --- | --- | --- | --- | --- | --- | --- | --- | --- | --- | --- | --- | --- | --- | --- | --- | --- | --- | --- | --- | --- | --- | --- |

| Table S6: Coefficients, standard errors, statistics, p-values, and confidence intervals for the Cox regression model with frailties that explains the time to the first SRS outbreak in a production cycle (Model 2).   \|  \| **Coefficient** \| **Lower CI95%** \| **Upper CI95%** \| **Exponentiated coefficient** \| **Exponentiated Lower CI95%** \| **Exponentiated Upper CI95%** \| \| --- \| --- \| --- \| --- \| --- \| --- \| --- \| \| Species: Rainbow trout \| 0.5 \| 0.21 \| 0.78 \| 1.64 \| 1.24 \| 2.18 \| \| Avg weekly Caligus counts (egg-laying females) \| 0.41 \| 0.29 \| 0.53 \| 1.5 \| 1.33 \| 1.69 \| \| Longitude (degrees) \| 0.18 \| 0.03 \| 0.34 \| 1.2 \| 1.03 \| 1.4 \| \| Number of mechanical damage high mortality events \| -0.13 \| -0.21 \| -0.04 \| 0.88 \| 0.81 \| 0.96 \| \| Number of environmental high mortality events \| -0.21 \| -0.29 \| -0.12 \| 0.81 \| 0.75 \| 0.89 \| \| Number of maturity-related high mortality events \| -0.3 \| -0.42 \| -0.17 \| 0.74 \| 0.65 \| 0.84 \| \| Number of amebiasis outbreaks \| -0.31 \| -0.49 \| -0.13 \| 0.74 \| 0.61 \| 0.88 \| \| Number of predator high mortality events \| -0.34 \| -0.43 \| -0.26 \| 0.71 \| 0.65 \| 0.77 \| \| Total Caligus high load reports (avg. female count > 1) \| -1.58 \| -1.73 \| -1.42 \| 0.21 \| 0.18 \| 0.24 \| \| Species: Coho salmon \| -2.49 \| -2.8 \| -2.17 \| 0.08 \| 0.06 \| 0.11 \| |
| --- | --- | --- | --- | --- | --- | --- | --- | --- | --- | --- | --- | --- | --- | --- | --- | --- | --- | --- | --- | --- | --- | --- | --- | --- | --- | --- | --- | --- | --- | --- | --- | --- | --- | --- | --- | --- | --- | --- | --- | --- | --- | --- | --- | --- | --- | --- | --- | --- | --- | --- | --- | --- | --- | --- | --- | --- | --- | --- | --- | --- | --- | --- | --- | --- | --- | --- | --- | --- | --- | --- | --- | --- | --- | --- | --- | --- | --- |
